# Potential of Pectins to Beneficially Modulate the Gut Microbiota Depends on Their Structural Properties

**DOI:** 10.3389/fmicb.2019.00223

**Published:** 2019-02-15

**Authors:** Nadja Larsen, Carlota Bussolo de Souza, Lukasz Krych, Thiago Barbosa Cahú, Maria Wiese, Witold Kot, Karin Meyer Hansen, Andreas Blennow, Koen Venema, Lene Jespersen

**Affiliations:** ^1^Department of Food Science, University of Copenhagen, Copenhagen, Denmark; ^2^Center for Healthy Eating and Food Innovation, Maastricht University – Campus Venlo, Maastricht, Netherlands; ^3^Department of Environmental Science, Aarhus University, Roskilde, Denmark; ^4^CP Kelco ApS, Lille Skensved, Denmark; ^5^Department of Plant and Environmental Sciences, University of Copenhagen, Copenhagen, Denmark; ^6^Beneficial Microbes Consultancy, Wageningen, Netherlands

**Keywords:** gut microbiota, pectins, structure-function relationship, TIM-2 colon model, short-chain fatty acids

## Abstract

Pectins are plant cell-wall polysaccharides which can be utilized by commensal bacteria in the gut, exhibiting beneficial properties for the host. Knowledge of the impact of pectins on intestinal bacterial communities is insufficient and limited to a few types of pectins. This study characterized the relationship between the structural properties of pectins and their potential to modulate composition and activity of the gut microbiota in a beneficial way. For this purpose we performed *in vitro* fermentations of nine structurally diverse pectins from citrus fruits and sugar beet, and a pectic derivative, rhamnogalacturonan I (RGI), using a TIM-2 colon model. The composition of microbiota during TIM-2 fermentations was assessed by 16S rRNA gene amplicon sequencing. Both general and pectin-specific changes were observed in relative abundances of numerous bacterial taxa in a time-dependent way. Bacterial populations associated with human health, such as *Faecalibacterium prausnitzii*, *Coprococcus*, *Ruminococcus*, *Dorea*, *Blautia*, *Oscillospira*, *Sutterella*, *Bifidobacterium*, *Christensenellaceae*, *Prevotella copri*, and *Bacteroides* spp. were either increased or decreased depending on the substrate, suggesting that these bacteria can be controlled using structurally different pectins. The main structural features linked to the pectin-mediated shifts in microbiota included degree of esterification, composition of neutral sugars, distribution of homogalacturonan and rhamnogalacturonan fractions, degree of branching, and the presence of amide groups. Cumulative production of the total short chain fatty acids and propionate was largest in fermentations of the high methoxyl pectins. Thus, this study indicates that microbial communities in the gut can be specifically modulated by pectins and identifies the features in pectin molecules linked to microbial alterations. This knowledge can be used to define preferred dietary pectins, targeting beneficial bacteria, and favoring more balanced microbiota communities in the gut.

## Introduction

Pectins are a part of daily diet consumed in form of fruits and vegetables. Besides, pectins are authorized as food additives and used as emulsifiers, gelling or stabilizing agents in yoghurts, jams, and other food products (Regulation (EC) (2008) No 1333/2008 of the European Parliament and of the Council of 16 December 2008 on food additives, 2008). Pectins are commercially produced from peel and pulp of fruits and vegetables, mainly, citrus fruits, sugar beet, and apples. Structural differences of pectins are defined by their source and production methods. The backbone of pectin molecules is composed of homogalacturonan or 1,4-linked α-D-galacturonic acid, occasionally substituted by Rha. The major side chain unit is RGI (“hairy” region), consisting of (1,4)-galacturonosyl and α-(1,2)-rhamnosyl chains with attached neutral sugars (galactan, arabinan or arabinogalactans) (Sila et al., 2009). The polygalacturonic acid in the backbone can be partially esterified with methyl groups. According to the DE, pectins are conventionally referred to as high methoxyl (HM) pectins (DE > 50%) and low methoxyl (LM) pectins (DE < 50%). Commercial LM pectins can also be amidated in order to achieve better gelling control (Chan et al., 2017).

Pectins are indigestible by human enzymes, however, can be easily degraded by commensal bacteria in the gut with production of SCFA and other metabolites (Fukunaga et al., 2003). Besides SCFA production, the beneficial effects of pectins include reduction of ammonia (Shinohara et al., 2010), delayed gastric emptying and improved Glc tolerance (Schwartz et al., 1988). Furthermore, pectins can induce gut immunity, improve intestinal integrity and mucosal proliferation, and favor adhesion of probiotic *Lactobacillus* strains to the epithelial cells (Fukunaga et al., 2003; Parkar et al., 2010; Larsen et al., 2018). The ability of pectins and POS to support the growth of specific bacterial populations has been described in several studies; however, there is some inconsistency in results. *In vitro* fermentations with pectins and POS stimulated various beneficial bacteria, including, bifidobacteria, lactobacilli, *Faecalibacterium prausnitzii*, *Roseburia* spp. and *Eubacterium rectale* (Manderson et al., 2005; Shinohara et al., 2010; Sulek et al., 2014; Gómez et al., 2016), though other studies reported unchanged or even decreased levels of bifidobacteria and *Roseburia* (Onumpai et al., 2011; Aguirre et al., 2014; Leijdekkers et al., 2014). Other bacterial taxa commonly increased by pectins, comprise *Bacteroides*, *Prevotella*, *E. rectale*/*C. coccoides* group and *Clostridium* spp. (Onumpai et al., 2011; Chen et al., 2013; Aguirre et al., 2014; Gómez et al., 2014; Leijdekkers et al., 2014; Reichardt et al., 2018). Species within genera *Bacteroides* and *Prevotella* are the primary pectin-degraders, possessing carbohydrate-active enzymes (CAZymes) within the PULs (Martens et al., 2011). The enzymes lyases, methylesterases, and acetylesterases facilitate the breakdown of pectins (Grondin et al., 2017). Variations in microbiota composition, enzyme capabilities and fermentation substrates, can explain inconsistencies between the studies in the effects of pectins and POS on microbial communities.

Recent studies indicate that functional properties of pectins in the gut might be linked to their structure, e.g., DE, distribution of free and methylated carboxyl groups within the polygalacturonic acid, molecular size, and sugar composition (Onumpai et al., 2011; Wicker et al., 2014). Pectins with lower DE and oligomeric size were preferentially metabolized and stimulated the growth of bifidobacteria in fecal fermentations and in mixed cultures (Dongowski et al., 2002; Olano-Martin et al., 2002; Li et al., 2016). In a recent study, Tian and coworkers reported the differences between the LM and HM pectins on the levels of fecal *Prevotella* and *Lactobacillus* in piglets (Tian et al., 2017). Different shifts in *Bifidobacterium*, *Bacteroides*/*Prevotella* and *C. coccoides*/*E. rectale* group were observed in fermentations of structurally different pectins from lemon and sugar beet (Gómez et al., 2016). However, up to now the impact on the gut microbiota has been studied for just a few types of pectins, and significance of their structural properties for microbiota shaping remains unclear. Previously, we showed that DE, net charge, DBr and molecular weight of pectins, were related to their ability to improve survival of probiotic strains at simulated gastro-intestinal conditions (Larsen et al., 2018). To reveal the structure-function relationship of pectins and the gut microbial community, we performed fermentations of structurally diverse pectins from citrus fruits and sugar beet using the TIM-2 colon model (TNO Innovation for Life, Netherlands) and characterized their potential to modify the gut bacterial populations in a beneficial way.

## Materials and Methods

### Pectins

Pectins (nine in total) were produced by CP Kelco (Denmark) from orange (P1 and P8), lemon (P2, P3, P6, and P9), lime (P5 and P7) and sugar beet (P4) using different extraction methods (harsh, mild, and differentially extracted) as shown in Table 1. Pectins P5 and P6, respectively, were chemically and enzymatically deesterified, and pectins P7 and P8 were amidated. Pectic derivative RGI (P10) was purified from lime.

**Table 1:** Pectins used in this study.

Pectin ID	Production methods	Source	DE, %^1^
P1	Harsh extracted	Orange	58.8
P2	Mild extracted	Lemon	70.0
P3	Differentially extracted	Lemon	74.7
P4	Harsh extracted	Sugar beet	59.3
P5	Harsh extracted chemically deesterified	Lime	11.4
P6	Mild extracted enzymatically deesterified	Lemon	31.8
P7	Harsh extracted amidated	Lime	28.8
P8	Harsh extracted amidated	Orange	29.5
P9	Harsh extracted	Lemon	35.6
P10	Rhamnogalacturonan I	Lime	nd


*^1^DE, degree of esterification; nd, not determined.*

### High Performance Anion Exchange Chromatography (HPAEC)

Monosaccharide composition of pectins (P1–P10) was determined by HPAEC equipped with a PA20 column and Pulsed Amperometric Detection (PAD, Dionex, CA, United States) as reported previously (Larsen et al., 2018). Monosaccharides, GalA, Ara, Rha, Gal, Glc, and Xyl were quantified from two independent HPAEC-PAD analyses. DBr of the rhamnosyl residues with neutral sugars side chains was calculated by the equation DBr(%) = 100% × Rha(%)/[(Ara(%) + Gal(%)]. The molar content of homogalacturonan (HG) and rhamnogalacturonan (RG) was estimated using equations: HG(%) = GalA(%) – Rha(%); RG(%) = 2Rha(%) + Ara(%) + Gal(%) (Yapo and Koffi, 2014). Differences between the pectins in the composition of monosaccharides were tested by the one-way ANOVA, Tukey’s *post hoc* test (Supplementary Table S1).

### TIM-2 Fermentations and Sample Collection

*In vitro* fermentations were performed using TIM-2 model of proximal colon model, providing fully anaerobic conditions, peristaltic movements and removal of metabolites during fermentation. The TIM-2 model has been validated and used as a predictive model for clinical trials (Venema, 2015). The microbiota inoculum consisted of an active, pooled fecal samples from 8 healthy Caucasian adults (male: *n* = 4, female: *n* = 4; age of 25–42 years). Fecal samples were maintained under anaerobiosis by using anaerobic packs (AnaeroGen^TM^, Oxoid, Cambridge, United Kingdom). The fecal microbiota was homogenized under anaerobic conditions, snap-frozen in liquid nitrogen and stored at -80°C before inoculation in TIM-2 (Aguirre et al., 2014). The TIM-2 colon model has been described previously, including operational units, maintenance, conditions of fermentation, and sample loading (Aguirre et al., 2014). Briefly, all units of the system were flushed with nitrogen prior to inoculation and throughout the experiments. Fermentations were performed at 37°C with the pH kept at 5.8 (or slightly above) by automatic titration with 2M NaOH. A 30 ml portion of culture homogenate was used to inoculate the units for each experiment. Following inoculation the microbiota was incubated for 16 h for adaptation to the new environment. During this period the basal SIEM, composed of complex carbohydrates, protein, ox-bile, Tween 80, vitamins, and minerals (Venema, 2015), was gradually introduced into the system in a total volume of 40 ml. After the adaptation, the culture was deprived from any medium for 2 h (starvation period). Afterward, the units were fed with SIEM containing 7.5 g pectin per day as the only carbohydrate source. The doses of pectin in TIM-2 fermentations was based on the amount of carbohydrates present in SIEM, and corresponded to approximately 250 g citrus fruit (grapefruit, lemon, and orange) with the typical content of pectins of 2.3–4.5% (Thakur et al., 1997). Luminal and dialysate samples were removed at the start of fermentation (0 h) and after 24, 48, 56, and 72 h fermentation for analyses of microbiota, SCFA and BCFA. Samples were snap-frozen in liquid nitrogen and stored (-80°C) until analysis. Fermentation of each substrate was performed in two independent TIM-2 experiments.

### DNA Purification

Microbiota samples from TIM-2 experiments were centrifuged (10,000 ×*g*, 10 min) and the fecal water discarded. The total bacterial DNA was isolated from the pellet using the PowerLyzer@PowerSoil DNA Isolation Kit (Qiagen Nordic, Denmark) according to manufacturer’s protocol with few modifications. Modifications included resuspension of fecal slurries in the Bead Solution, transfer to the PowerLyser^®^ Glass bead tubes and heating the tubes at 65°C for 10 min before the bead beating step. The homogenization was performed at speed setting of 6.5 m/s for 3 cycles, 30 s each with 30 sec interval (FastPrep-24^®^, MP Biomedicals, Solon OH, United States). The concentration and purity of DNA was determined using NanoDrop 2000 Spectrophotometer (Thermofisher Scientific, Denmark). The DNA concentration was typically in the range of 20–100 ng/μl and A260/A280 ratios of 1.8–2.1.

### 16S rRNA Gene Amplicon Sequencing and Data Processing

The DNA library for amplicon sequencing was prepared according to Williams et al. (2017). In brief, the V3 region (∼190 bp) of the 16S rRNA gene was PCR amplified using AccuPrime SuperMix II (Life Technologies, CA, United States) and primers NXt_388F and NXt_518R compatible with Nextera Index Kit (Illumina). Amplification steps included initial denaturation at 95°C for 2 min, 33 cycles of denaturation at 95°C for 15 s, annealing of primer at 55°C for 15 s and elongation at 68°C for 30 s. The second PCR (PCR II) was performed using Phusion High-Fidelity PCR Master Mix (ThermoFisher Scientific, MA, United States) and primers P5 and P7 (Nextera Index Kit) to incorporate adapters and tags in the PCR product. The PCRII setup included 13 cycles of denaturation at 98°C for 10 s, annealing of primer at 55°C for 20 s and elongation at 72°C for 20 s, followed by an extension at 72°C for 5 min. Amplifications were performed in a Veriti 96-well thermal cycler (Applied Biosystems, United States). The amplified fragments with adapters and tags were purified using AMPure XP beats (Beckman Coulter Genomic, CA, United States). Tag-encoded 16S rRNA gene sequencing was performed using Illumina NextSeq 550 Sequencing System using the 2 × 150 cycles MID V2 kit (Illumina, CA, United States). The raw dataset, containing pair-ended reads with corresponding quality scores, were trimmed, merged, clustered [operational taxonomic units (OTU) with 97% similarity], filtered from chimeric sequences using UPARSE and taxonomically assigned using the GreenGenes database (version 12.10) (Williams et al., 2017).

### Analysis of Sequencing Data and Statistic

Quantitative insight into microbial ecology (QIIME) software package (version 1.8.0) was used for subsequent analysis steps (Caporaso et al., 2010). The sequences were classified using Greengenes database (version 13.8) as a reference 16S rRNA gene database (Mcdonald et al., 2012). Alpha-diversity was evaluated by the number of observed species and Chao1 (97% similarity OTUs) computed for each OTU table rarefied to 10,000 sequences per sample, based on the lowest number of sequences produced per sample. Good’s coverage estimates were over 98.2% across the samples (data not shown), suggesting that sampling depth of 10,000 reads was sufficient to capture majority of the OTUs. Differences in alpha-diversity over time were tested using Student’s *t*-test, employing the non-parametric (Monte Carlo) method (999 permutations). Differences between the pectins in Chao1 indices were assessed by a one-way ANOVA with LSD *post hoc* test using data obtained in two independent experiments. Community differences (beta-diversity) were revealed by weighted and unweighted UniFrac distances matrices computed from the rarefied OTU tables and evaluated by analysis of similarities (ANOSIM). PCoA plots were generated with Jackknifed Beta Diversity workflow using 10 subsampled OTU tables. Group differences in microbial community structure were assessed by hierarchical clustering of OTUs (97% similarity), applying UPGMA (unweighted pair group method using arithmetic averages) algorithm and visualized using R-studio software heatmap2 (cut-off 0.01%). Statistical differences in bacterial abundances were evaluated for pooled data set (48, 56, and 72 h from two independent experiments, *n* = 6 for each substrate) compared to time 0 h (baseline, *n* = 20) by the non-parametric Wilcoxon Rank Sum test combined with Bonferroni multiplicity correction, using 0.05 as significance level. Correlation between the relative abundances of OTUs and the structural characteristics of nine pectins (P1–P9) was evaluated with the Spearman rank correlation test implemented in the otu_category_significance.py script (QIIME 1.8.0). Statistical significance was evaluated from the conservative FDR corrected *p*-values for multiple comparisons (FDR-adjusted, *p* < 0.05).

### Analysis of Short Chain Fatty and Branched Chain Fatty Acids

The amounts of SCFA and BCFA were determined in the lumen and dialysate TIM-2 samples by Brightlabs (Venlo, Netherlands). Samples were centrifuged (10,000 ×*g*, 20 min) and the supernatants were diluted with 1.5 mM sulfuric acid. The samples (10 μl) were injected onto an 883 Basic IC plus ion-chromatography system with suppressed conductivity detection used in positive mode (Metrohm, Herisa, Switzerland). The acids were separated by isocratic elution on ICsep ION300 Ion exclusion column (300 mm × 7.8 mm, 7 μm particle size) and Metrosep RP2 Guard column (Transgenomic, New Haven, CT, United States) using 1.5 mM sulfuric acid as a mobile phase. Samples were eluted at a flow rate of 0.4 ml/min with a pressure of 5.5 MPa at 65°C. Acetic, propionic, butyric, iso-butyric, and iso-valeric acids were used as calibration standards (Sigma Chemical, St. Louis, MO, United States). Statistical differences between the pectins in SCFA and BCFA production over time were assessed by one-way ANOVA, Tukey’s *post hoc* test. Differences between the high methoxyl pectins (P1, P2, P3, and P4) and LM pectins (P5, P6, P7, P8, and P9) in fatty acids production were evaluated by the two-sided Wilcoxon Rank Sum test. Correlation between the relative abundances of OTUs and cumulative production of fatty acids was assessed with the Spearman rank correlation test (QIIME 1.8.0) and statistical significance was evaluated from the FDR corrected *p*-values. In all tests differences were assumed to be statistically significant at *p*-values < 0.05.

## Results

### Composition and Structural Properties of Pectins

In total nine pectins (P1–P9) and RGI (P10), were used as substrates for microbiota fermentation in TIM-2 colon model (Table 1). The structural and physicochemical properties of pectins have been described previously (Larsen et al., 2018). The composition of monosaccharides referred to in this study is presented in Supplementary Table S1. Pectins P1, P2, P3, and P4 were high methoxyl pectins with highest DE of 70.0 and 74.7% in P2 and P3, respectively; others were LM pectins with DE between 11.4 and 35.6%, lowest in P5. The major neutral sugars in pectin molecules were galactose (Gal, 9.1–30.7%), arabinose (Ara, 0.8–16.7%), rhamnose (Rha, 1.7–3.7%) and Glc (Glc, 0.7–8.7%), while Xyl was found in lesser amounts (<1%). Content of GalA and HG was largest in pectins P2, P3, P6, and P9 from lemon (GalA of 70.7–83.0% and HG of 69.6–81.2%) and lowest in sugar beet pectin (P4) (46.9% of GalA and 43.9% of HG). Additionally, sugar beet pectin was characterized by relatively higher fractions of RG (50.7%) and neutral sugars (Gal, Ara, and Rha). RGI (P10) differed from pectins by low percentage of GalA (11.0%) and Gal (7.9%), and high fraction of Ara (69.9%), Rha (6.6%), and Xyl (1.8%). Estimated DBr of rhamnosyl residues with neutral sugars side chains varied between 7.4% (P3) and 19.2% (P6).

### Alpha-Diversity of Microbiota

**FIGURE 1:**
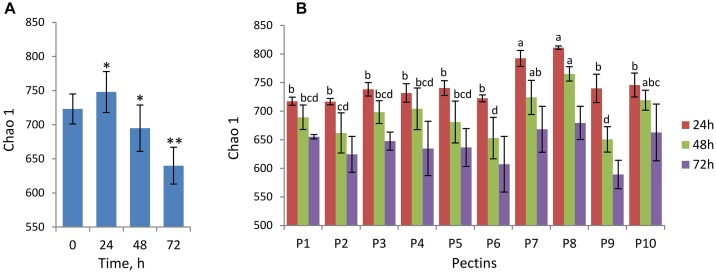
Analysis of alpha-diversity over fermentation time and between the pectins presented by Chao1 index. Microbiota was analyzed using Illumina NextSeq 550 Sequencing System. **(A)** Chao1 at baseline (0 h) and after 24, 48, and 72 h fermentation computed for combined samples (*n* = 20 for each time point). Asterisks denote the values significantly different from time 0 h (non-parametric Student’s *t*-test, ^∗^*p* < 0.05, ^∗∗^*p* < 0.01). **(B)** Chao1 after 24 h (brown columns), 48 h (green columns), and 72 h (purple columns) fermentation of pectins P1–P10, presented by means and standard deviation (bars) from two biological replicates. Superscripts (a, b, c, and d) show significant differences between the substrates at time-points 24 and 48 h (one-way ANOVA, LSD *post hoc* t test, *p* < 0.05). Chao1 indices at 72 h were not significantly different (letters not shown).

Changes in alpha-diversity of microbiota over time are presented by Chao1 indices (Figure 1A,B) and observed species number (Supplementary Figure S1). Both Chao1 and the number of observed species increased significantly after 24 h fermentation (Chao1of 746 ± 30) compared to baseline (Chao1of 723 ± 22) and, afterward, decreased gradually reaching the lowest values at the end of fermentation (Chao1of 640 ± 27 after 72 h) (Figure 1A). Fermentations of pectins P7 and P8 generally produced more diverse microbiota communities after 24–48 h compared to other substrates (Chao 1 of 724 ± 30 and 765 ± 12 after 48 h, respectively), whereas diversity in P6 and P9 fermentations was lowest (Chao 1 of 653 ± 36 and 651 ± 22 after 48 h, respectively) (Figure 1B). Differences between the substrates in alpha-diversity indices after 72 h fermentation were insignificant.

### Group Diversity of Microbiota

**FIGURE 2:**
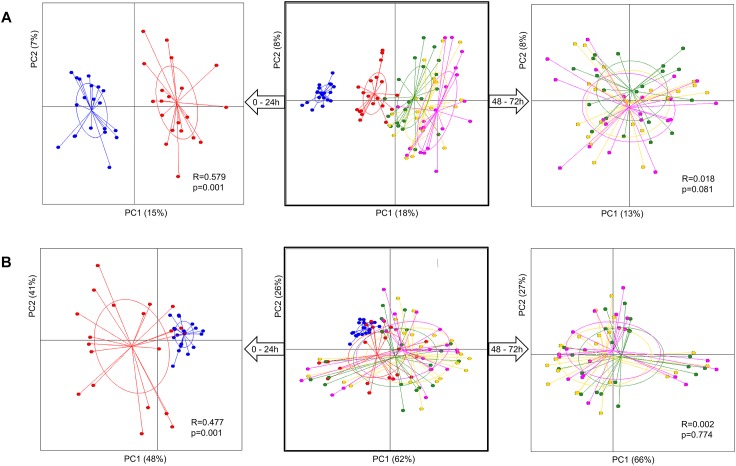
Principal coordinate analysis (PCoA) plots, showing group diversity over time, 0–72 h (middle plots), 0–24 h (plots to the left), and 48–72 h (plots to the right), assessed by unweighted **(A)** and weighted **(B)** UniFrac distance matrices. Samples are colored according to the time-points: blue dots – 0 h, red dots – 24 h, green dots – 48 h, yellow dots – 56 h, and pink dots – 72 h. Samples were collected from two independent fermentations of each substrate (P1–P10) and analyzed for microbiota composition using Illumina NextSeq 550 Sequencing System. Group differences were tested by ANOSIM as shown by R-values and *p*-values. The samples are plotted on the first two principal coordinates PC1 and PC2. The ellipse center indicates group means for each time-point.

**FIGURE 3:**
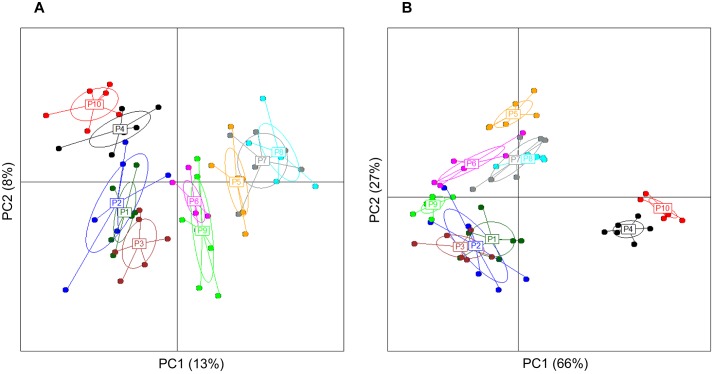
Principal coordinate analysis, showing group diversity between the pectins (P1–P10), as determined by unweighted **(A)** and weighted **(B)** UniFrac distance matrices. PCoA plots were constructed for the samples collected after 48, 56, and 72 h fermentation from two independent experiments. Microbiota composition was analyzed using Illumina NextSeq 550 Sequencing System. The ellipse center indicates group means. The samples are plotted on the first two principal coordinates PC1 and PC2.

Analysis of group diversity (beta-diversity) across the sampling times (Figure 2A,B) and the pectins (Figure 3A,B) is presented by the PCoA plots using unweighted and weighted UniFrac distance matrices. Unweighted UniFrac matrix is based on the presence/absence of bacterial species, accounting for both abundant and rare lineages; while weighted matrix relies on the absolute proportions, and it is most sensitive to detect the changes in dominant taxa. Major shifts in microbial diversity occurred after 24 h fermentation, as seen from both unweighted and weighted PCoA and confirmed by ANOSIM (*p* = 0.001) (Figure 2A,B). Differences in group diversity between the time-points 48, 56, and 72 h were insignificant, especially based on weighted UniFrac (*R* = 0.002, *p* = 0.774; Figure 2B), indicating stabilization of microbial communities after 48 h fermentation. Variation between the pectins could be best explained by the weighted PCoA (PC1 66% and PC2 27%), suggesting the major effect of abundant bacterial taxa for group differentiation (Figure 3B). Pectins P4 and P10 (group 1) were clearly separated from other substrates along the PC1 (weighted PCoA, Figure 3B). Other distinct groups combined pectins P1, P2, and P3 (group 2), and pectins P7 and P8 (group 3) (Figure 3A,B).

### Effect of Pectins on Microbiota Composition

**FIGURE 4:**
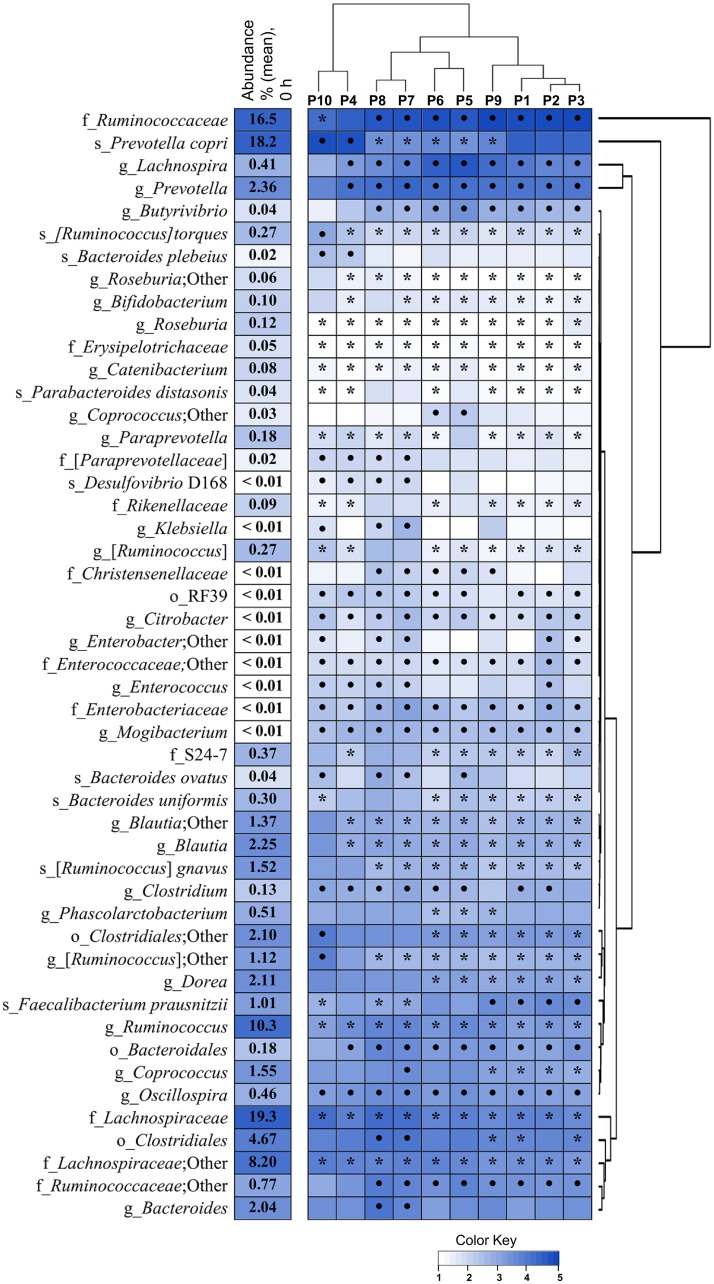
Changes in relative abundances of species level OTUs in TIM-2 fermentations of pectins (P1–P10). Microbiota composition was analyzed using Illumina NextSeq 550 Sequencing System. The heatmap and dendograms were generated using UPGMA algorithm and R-studio software (heatmap2). Abundances at fermentation start (0 h) are shown by numbers (means from two independent experiments, *n* = 20). Statistical differences after 48 h fermentation were evaluated for combined data (48, 56, and 72 h) obtained for each pectin in two independent experiments (*n* = 6), using the Wilcoxon Rank Sum test and Bonferroni multiplicity correction (*p* < 0.05). Significant increases in relative abundances compared to baseline (0 h) are marked by dots (∙) and decreases by asterisks (^•^). Taxa denoted as “Other” indicate ambiguity in the assignment; taxa in square brackets indicate a proposed taxonomy. The color key presents the log-transformed mean values.

In total 1218 OTUs were assigned to more than 150 bacterial taxa at species-level. Significant changes in bacterial abundances after 48 h fermentation of pectins (P1–P10) as shown by the heatmap in Figure 4. Regarding microbiota similarity between 48 and 72 h fermentation (Figure 2A,B), statistical differences between the pectins were evaluated for the combined data. The hierarchical clustering of individual samples and the relative abundances of species-level OTUs (cut-off 0.01%) at baseline (0 h) and after 48, 56, and 72 h fermentation are presented in Supplementary Figure S2. Four bacterial taxa were prevalent at fermentation start (64.5% in total), including family *Lachnospiraceae* (19.3%), family *Ruminococcaceae* (16.5%) and genus *Ruminococcus* (10.4%) within phylum *Firmicutes*, and species *Prevotella copri* (18.3%) within phylum *Bacteroidetes*. Other 26 common bacterial populations, belonging to phyla *Firmicutes* and *Bacteroidetes*, were found in moderate numbers (0.1–10%); among them, genera *Blautia*, *Dorea*, *Coprococcus*, *Ruminococcus*, *Bacteroides*, *Prevotella* and species *F. prausnitzii*. Less abundant phyla at baseline were *Actinobacteria* (0.41%), presented mainly by genera *Bifidobacterium* and *Collinsella*, phylum *Proteobacteria* (0.09%), presented by *Desulfovibrio* spp., *Sutterella* and *Enterobacteriaceae*, and phylum *Tenericutes* (0.02%), comprising *Anaeroplasma* and order RF39.

Cluster analysis revealed grouping of pectins into several clusters after similarity in microbiota profiles (Figure 4 and Supplementary Figure S2). A distinct cluster combined pectins P4 and P10, which separation was likely driven by comparatively higher numbers of *P. copri*, *Bacteroides plebeius*, *Ruminococcus gnavus* and lower proportions of *Ruminococcaceae* (Figure 4). Pectins P7 and P8 were merged into another cluster, having increased levels of order *Clostridiales*, *Coprococcus, Bacteroides*, family *Christensenellaceae* and *Proteobacteria* (*Disulfovibrio* D168, *Klebsiella*, and *Enterobacter*), along with reduction in *F. prausnitzii*. Grouping of pectins P1, P2, P3, and P9 was characterized by lower abundances of *Coprococcus* and *Paraprevotellaceae*, and relatively high numbers of *F. prausnitzii*. Clustering of P5, P6 can be partially explained by higher proportions of *Lachnospira*, *Butyrivibrio*, *Phascolarctobacterium* and *Coprococcus*, along with lower fraction of *P. copri*. Bacterial populations generally reduced by all pectins, included *Ruminococcus* spp., genera *Blautia*, *Roseburia*, *Catenibacterium*, *Bifidobacterium*, *Paraprevotella*, and families *Lachnospiraceae* and *Erysipelotrichaceae*. Concurrently, abundances of *Lachnospira*, *Oscillospira*, *Clostridium*, *Butyrivibrio, Prevotella*, family *Ruminococcaceae* and order *Bacteroidales*, were mostly increased. Additionally, the rare taxa, i.e., genus *Mogibacterium*, family *Enterobacteriaceae* (genus *Citrobacter*) and order RF39 (phylum *Tenericutes*) were stimulated in all fermentations, though to a different extent (Figure 4).

### Correlation Between Bacterial Abundances and the Structural Properties of Pectins

**FIGURE 5:**
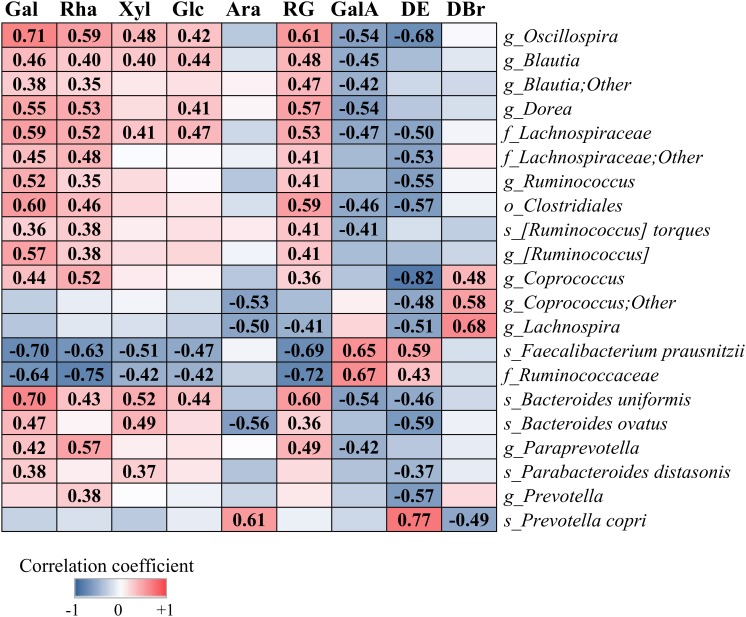
Correlation between the relative abundances of bacterial taxa and the structural characteristics of pectins (P1–P9), i.e., content of galactose (Gal), rhamnose (Rha), xylose (Xyl), glucose (Glc), arabinose (Ara), galacturonic acid (GalA), rhamnogalacturonan (RG), degree of branching (DBr), and degree of esterification (DE). Analysis was performed by the Spearman rank correlation test using combined data (48, 56, and 72 h), obtained for each pectin from two independent experiments. Significant correlation is indicated by the correlation coefficient (FDR-adjusted, *p* < 0.05).

Figure 5 shows correlation between the relative abundances of species-level OTUs and characteristics of pectins, i.e., content of monosaccharides, GalA, RG fraction, DE and DBr. The levels of *Oscillospira*, *Blautia*, *Dorea*, *Ruminococcus*, *Coprococcus*, *R. torques*, *Lachnospiraceae* and *Clostridiales* within phylum *Firmicutes*, and *Paraprevotella*, *B. uniformis*, *B. ovatus*, *P. distasonis* and *Prevotella* within phylum *Bacteroidetes*, correlated significantly (*r* = 0.35–0.71, *p* < 0.05) or showed a tendency to increase with higher fractions of simple sugars (Gal, Rha, Xyl, and Glc) and lower content of GalA and DE values. Species *F. prausnitzii* and family *Ruminococcaceae* showed positive correlation with the sugar residues, GalA and DE (*r* = 0.42–0.72, *p* < 0.01), in contrast to bacterial taxa above. Abundance of *P. copri* correlated positively with Ara (*r* = 0.61, *p* < 0.001) and DE (*r* = 0.77, *p* < 0.001) and negatively with DBr (*r* = 0.49, *p* < 0.01). Additionally, genera *Coprococcus* and *Lachnospira* correlated positively with DBr (*r* = 0.48–0.68, *p* < 0.01) and negatively (*r* = 0.48–0.82, *p* < 0.01) with DE and Ara.

### Production of Short Chain and Branched Chain Fatty Acids

The cumulative production of SCFA (acetate, propionate and n-butyrate) after 72 h fermentation is shown in Table 2. The amounts of acetate and total SCFA were not different between the pectins, comprising 81–95 mmol and 131–162 mmol, respectively. Production of propionate was largest for sugar beet pectin (P4) (43.3 mmol) and RGI (P10) (47.6 mmol), and differed significantly from the citrus pectins P2, P5, P6, P7, and P9 (24.2–30.1 mmol). Generally, the HM pectins (P1, P2, P3, and P4) generated higher amounts (*p* < 0.05) of propionate and total SCFA compared to LM pectins (P5, P6, P7, P8, and P9). Production of butyrate was lowest in fermentations of P10 (19.3 mmol) and statistically different from pectins P1, P2, P3, and P9 (27.9–32.9 mmol). The lowest acetate to propionate ratios (2.0–2.4) were determined for P4, P1, and P10 and highest (3.2–3.6) for P5 and P6 fermentations. Production of total BCFA, i-butyrate and i-valerate were slightly lower in RGI fermentations, though not significantly different from other treatments (Supplementary Table S2). The amount of propionate throughout fermentation (24–72 h) correlated positively with *P. copri*, *R. torques* and unclassified *Clostridiales*, and, negatively, with genera *Coprococcus*, *Butyrivibrio* and *Lachnospira* (Table 3). Production of butyrate decreased with increasing abundances of *B. ovatus* and *Bacteroides* after 48 h fermentation. Correlation between other bacterial taxa and SCFA was insignificant.

**Table 2:** Cumulative production of acetate, propionate, n-butyrate and total short chain fatty acids (SCFA) after 72 h fermentation of pectins in TIM-2 colon model^1^.

Pectin ID	Acetate, mmol	Propionate, mmol	n-butyrate, mmol	Total SCFA, mmol	Ratio Acetate/Propionate
P1	86.6 (1.6)^a^	36.1 (2.3)^bc^	32.9 (2.9)^a^	155.6 (6.7)^a^	2.4 (0.1)^def^
P2	85.0 (0.6)^a^	30.1 (5.4)^cd^	28.8 (2.9)^ab^	143.8 (1.9)^a^	2.9 (0.5)^abcd^
P3	86.8 (3.2)^a^	32.7 (1.5)^bcd^	27.9 (1.5)^ab^	147.5 (3.2)^a^	2.7 (0.1)^cdef^
P4	95.3 (0.2)^a^	43.3 (0.2)^ab^	24.0 (1.5)^bc^	162.6 (1.5)^a^	2.2 (0.1)^ef^
P5	82.7 (2.7)^a^	24.3 (1.5)^d^	23.9 (0.5)^bc^	131.0 (3.7)^a^	3.4 (0.1)^ab^
P6	87.4 (5.6)^a^	24.2 (0.6)^d^	26.7 (0.9)^abc^	138.3 (3.6)^a^	3.6 (0.3)^a^
P7	93.4 (11.4)^a^	30.0 (1.9)^cd^	24.1 (2.2)^bc^	147.5 (11.2)^a^	3.1 (0.2)^abcd^
P8	92.3 (10.1)^a^	32.6 (3.4)^bcd^	21.9 (3.5)^bc^	146.7 (17.0)^a^	2.8 (0.1)^bcde^
P9	80.8 (2.4)^a^	25.4 (2.1)^cd^	28.2 (1.3)^ab^	134.3 (5.7)^a^	3.2 (0.2)^abc^
P10	92.5 (2.3)^a^	47.6 (8.0)^a^	19.3 (1.1)^c^	159.4 (11.4)^a^	2.0 (0.3)^f^
HM/LM^2^	88.4/87.3^ns^	35.6/27.3^∗∗^	28.4/25.0^ns^	152.4/139.6^∗^	2.55/3.23^∗∗^


*^1^Mean values and SD (in brackets) in lumen and dialysate (combined data) from two independent TIM-2 experiments. Total SCFA is a sum of acetic, propionic and butyric acids. Different superscripts within a column (a, b, c, d, e, and f) indicate significant differences between the substrates (one-way ANOVA Tukey’s *post hoc* test, *p* < 0.05). Pectins are denoted by codes described in Table 1. ^*2*^Amounts of SCFA (means) produced by HM pectins (P1, P2, P3, and P4) relative to LM pectins (P5, P6, P7, P8, and P9). Significant differences between the HM and LM pectins were determined by the two-sided Wilcoxon rank sum test (^∗^*p* < 0.05; ^∗∗^*p* < 0.01; ns, insignificant).*

**Table 3:** Correlation between the relative abundances of bacterial taxa and cumulative production of the short chain fatty acids (SCFA) propionate and butyrate after 24, 48. and 72 h fermentation of pectins in TIM-2 colon model.

SCFA	Bacterial taxa	Correlation coefficient, r^1^		
		**24 h**	**48 h**	**72 h**
Propionate	*s_Prevotella copri*	0.80***	0.81***	0.73***
	*g_[Ruminococcus];Other*	0.48*ns*	0.71**	0.61*
	*s_[Ruminococcus] torques*	0.62*	0.64*	0.67*
	*o_Clostridiales;Other*	0.39*ns*	0.61*	0.62*
	*f_Ruminococcaceae;Other*	–0.66*	–0.69**	–0.51
	*g_Coprococcus;Other*	–0.73**	–0.32*ns*	–0.72**
	*g_Butyrivibrio*	–0.64*	–0.73**	–0.61*
	*g_Lachnospira*	–0.70**	–0.80***	–0.77***
Butyrate	*s_Bacteroides ovatus*	–0.53*ns*	–0.79**	–0.66*
	*g_Bacteroides*	–0.57*ns*	–0.64*	–0.40*ns*


*^1^Spearman rank correlation coefficient (r); asterisks denote significant correlation (FDR-adjusted, ^∗^*p* < 0.05; ^∗∗^*p* < 0.01; and ^∗∗∗^*p* < 0.001); ns, insignificant.*

## Discussion

### Increase in Bacterial Abundances Was Related to Species-Specific Utilization of Pectins

In this study we conducted TIM-2 fermentations with diverse pectins to investigate the relationship between the structural properties of pectins and the changes in microbiota composition. Enrichment or reduction of bacterial populations during fermentation would depend on their ability to degrade pectins, and/or utilize POS and other metabolites (cross-feeding interactions). Among bacterial taxa increased in this study, the members of *Bacteroidales*, genera *Prevotella*, *F. prausnitzii* and family *Enterobacteriaceae*, have been reported as fiber- or pectin-degraders. In addition to the primary pectin-degraders *Bacteroidetes* and *Prevotella*, described in introduction, structure and activity of a large variety of pectinases have been characterized within the members of *Enterobacteriaceae* (*Erwinia*, *Yersinia*, and *E. coli*) and *Clostridium* species of intestinal origin (Nakajima et al., 2002; Abbott and Boraston, 2008). Ability of intestinal isolates of *Clostridium*, *Bacteroides*, *Enterococcus*, and *F. prausnitzii* to utilize pectins was previously confirmed in culture growth experiments (Olano-Martin et al., 2002; Lopez-Siles et al., 2012). Besides, *Oscillospira* and *Clostridium* species were found to be associated with degradation of plant material and enhanced by plant fiber content in human diet (Mackie et al., 2003; Tims et al., 2013). Interestingly, shifts in related species of *Bacteroides ovatus*, *B. plebeius* and *B. uniformis* in this study were substrate dependent, which can be explained by the species-specific activity of pectin-degrading enzymes and the hierarchical preference of substrate utilization. Accordingly, Tuncil et al. (2017) demonstrated that human gut symbionts *B. thetaiotamicron* and *B. ovatus*, grown together on POS, had inverse growth profile, concurrently with the different expression pattern of glycan utilization genes. Preferential utilization of metabolites from pectin degradation can be expected, regarding the highly competitive environment in fecal fermentations. High increase in genera *Butyrivibrio* and *Lachnospira*, previously shown incapable to degrade pectins (van Laere et al., 2000; Flint et al., 2012), might be linked to metabolic cross-feeding interactions between the members of the fecal microbiota.

### Bacterial Populations Associated With Human Health Were Selectively Stimulated by Pectins

Pectin fermentations affected abundances of various bacterial taxa associated with microbiota dysbiosis in human diseases, such as obesity and IBD. Thus, it was previously shown that obesity-related bias in the gut microbiota included higher levels of *Blautia*, *Eubacterium*, *Roseburia, Dorea* and *Ruminococcus*, along with reduction of *F. prausnitzii*, *Oscillospira*, *Christensenellaceae*, *Prevotella, Bacteroides* and genera within *Proteobacteria* (Nadal et al., 2009; Furet et al., 2010; Tims et al., 2013; Verdam et al., 2013; Goodrich et al., 2014; Kasai et al., 2015). Besides, *R. torques* and *R. gnavus*, decreased in this study, were found in higher numbers in IBD patients (Png et al., 2010). Notably, shifts in *F. prausnitzii*, *Coprococcus*, *B. ovatus, B. plebeius, P. copri* and *Sutterella* were strongly dependent on the feed, suggesting that these species can be modulated by specific pectins. Among them, *F. prausnitzii*, is commonly referred to as a marker for intestinal health, exhibiting anti-inflammatory effects in the gut (Walters et al., 2014). This study indicated that stimulation of *F. prausnitzii* could be achieved by fermentation of high methoxyl pectins (P1–P3) rather than LM pectins (P7 and P8). In their turn, LM citrus pectins were efficient to decrease proportions of *P. copri*, a microbe associated with induced insulin resistance in mice and rheumatoid arthritis in humans (Pedersen et al., 2016; Pianta et al., 2017); and to increase the levels of *Coprococcus* linked to reduced severity of IBS in humans (Tap et al., 2017).

### Production of Propionate, Butyrate and Total SCFA Differed Between the Pectins

The overall production of propionate was highest in RGI fermentations and in fermentations of high methoxyl pectins (especially P4) and correlated positively with the relative abundances of *P. copri*, *Ruminococcus* spp, and unidentified *Clostridiales*, suggesting that these species were able to generate propionate from pectin fermentation products. Supporting our results, Gulfi and coworkers reported that HM pectins had a tendency to produce larger amounts of propionic acid in batch fecal fermentations, compared to LM pectins (Gulfi et al., 2005). Additionally, the ability of *Prevotella* and *Ruminococcus* to produce propionate was recently confirmed by genomic analysis of colonic anaerobes combined with growth experiments (Qin et al., 2010; Reichardt et al., 2014; Chen et al., 2017). Production of propionate by *Ruminococcus* is known to be enhanced in the presence of Rha and fucose (Reichardt et al., 2014), which are the common structural units of pectin molecules. Consequently, increased production of propionate in fermentations of sugar beet pectin (P4) and RGI (P10) might be related to the relatively higher content of Rha and other neutral sugars. Interestingly, the acetate to propionate ratios were found to be reduced in RGI and the high methoxyl pectin group, lowest for P4 and harsh extracted pectin from orange (P1). Lower acetate/propionate ratios have been associated with an anti-cholesterolemic effect and reduction of cardiovascular disease risk, and generally considered as beneficial (den Besten et al., 2013). The lowest amount of butyrate was detected in RGI fermentations, which might be explained by decreased levels of *F. prausnitzii*, a predominant butyrate producer in the gut (Louis and Flint, 2017). The negative correlation between butyrate and the numbers of *Bacteroides* spp. was, probably, related to their inability to produce butyrate (Louis and Flint, 2017). Production of SCFA in fermentations of citrus pectins (P1–P3 and P5–P9) was not statistically different, despite the differences in microbiota composition. This effect was presumably caused by the functional redundancy of microbial populations and the metabolic cross-feeding interactions (Reichardt et al., 2018).

### Changes in Microbiota Were Related to the Structural Features of Pectins

Shifts in bacterial abundances in TIM-2 fermentations were related to the structural features of pectins. We identified at least five factors essential for microbiota shaping: (i) DE of polygalacturonic acid, (ii) composition of neutral sugars, (iii) distribution of HG and RG fractions, (iv) DBr, and (v) modification of pectic backbone, e.g., by amidation. DE is probably the most important parameter, as seen from the beta-diversity analysis and the correlation of bacterial taxa with DE. So far, only few studies indicated a link between the DE in pectins and microbiota composition. In agreement with our results, enrichment in *Prevotella* spp. was found in colonic microbiota of pigs fed with LM pectins, while genus *Bacteroides* was increased in fecal batch fermentations of LM pectin contrary to HM pectin (Dongowski et al., 2002; Tian et al., 2017). Results of the correlation analysis (Figure 5) pointed out on the possibility of differential stimulation of bacterial populations using pectins with different sugar content, especially Rha and Gal. Thus, correlation of *F. prausnitzii* with the major sugars (negative) and GalA/DE (positive) suggested, that HM pectins with high fraction of HG over RG would be preferable for growth of this beneficial microbe. Interestingly, the residue Ara differed from other sugars, correlating positively with *P. copri* and negatively with *Coprococcus* and *Lachnospira*. Both *Lachnospira* spp. and *P. copri* possess α-arabinofuranosidase activity (Dodd et al., 2011), having potential to degrade arabinan side chains in pectin molecules. Thus, inverse correlation is most probably linked to the differences between bacterial species in activity of arabinanolytic enzymes and the metabolic hierarchy as discussed above. Unexpectedly, we observed similarity in microbiota profiling between the structurally different substrates, i.e., sugar beet pectin and RGI. This similarity might be associated with HG/RG distribution, as both sugar beet pectin and RGI were distinguished by the low HG (GalA) and high RG content compared to other pectins. Likewise, grouping of the LM pectin P9 together with the high methoxyl pectins (P1 and P2) might be related to high percentage of HG in these substrates. Similarity between pectins P7 and P8 is most probably attributed to amidation of the C-6 uronate groups in addition to the equally low DE values. It is well-known that structural features of pectins affect their rheological behavior and functional properties, e.g., gelling capacity, viscosity, molecular conformation, and solubility (Sila et al., 2009). We suppose that these properties might have an impact on substrate-bacterial interactions and pectin utilization in microbiota fermentations. Furthermore, carboxyl groups in the polygalacturonic chains can be involved in electrostatic interactions and, together with amide groups, in hydrogen bonding. In the previous study, we observed that the surface charge (or zeta-potential) of the LM pectins, was lower than that of HM pectins, due to the higher fraction of non-esterified carboxyl groups (Larsen et al., 2018). Lower zeta-potential in LM pectins indicated stronger electrostatic repulsion upon interactions with bacterial cell wall groups (hydroxyl, carboxyl, etc.) negatively charged at neutral pH (Jiang et al., 2004). High DBr and RG fraction can either provide steric hindrance or contribute to hydrophobic interactions with non-polar groups in bacterial cell walls. Involvement in the different types of interactions can, probably, explain the opposite correlation found for DE (and GalA) compared to DBr and neutral sugars with bacterial taxa in this study.

In conclusion, this study provided evidence that modulation of the gut microbiota by pectins depended on their structural features. We identified specific bacterial taxa which abundances were differentially affected by pectins and proposed the main factors, linked to differences in microbiota composition. Understanding of the interplay between the gut commensals and the structural properties of pectins is essential to predict physiological effects of ingested pectins and to provide ideas for development of pectin-containing dietary fibers, targeting beneficial bacteria to facilitate more balanced microbiota profiles. It would be relevant to verify our findings in relation to other gut microbiotas, e.g., from diseased subjects. Additionally, further comparative *in vitro* and *in vivo* studies with structurally diverse pectins and their derivatives are needed to achieve the detailed knowledge of structure-function relationship of pectins in the gut.

## Availability of Data

The metadata have been deposited in the European Nucleotide Archive (ENA) database [Accession No. PRJEB25646]. Mapping file, explaining the sample names in deposited metadata, is presented in Supplementary Table S3.

## Ethics Statement

Studies using fecal donations from healthy volunteers do not require medical ethical committee approval in Netherlands, since they are considered as non-invasive. Nevertheless, all participants provided informed consent. Results in this manuscript are referred to a pool fecal inoculum and do not directly refer to a particular person.

## Author Contributions

LJ, NL, KV, and AB conceived, planned and coordinated the study. KV and CBS performed the fermentations, sample collection, and SCFA analysis. NL conducted to microbiota analysis and wrote manuscript. LK and WK performed the sequencing and bioinformatics. KH and TBC participated in production and characterization of pectins. MW advised in statistical analysis. All authors read and approved the final manuscript.

## Conflict of Interest Statement

The authors declare that the research was conducted in the absence of any commercial or financial relationships that could be construed as a potential conflict of interest.

## Abbreviations

ANOVA: analysis of variance
Ara: arabinose
BCFA: branched chain fatty acids
DBr: degree of branching
DE: degree of esterification
DNA: deoxyribose nucleic acid
FDR: false discovery rate
Gal: galactose
GalA: galacturonic acid
Glc: glucose
HM: high methylated
HPAEC: high performance anion exchange chromatography
IBD: inflammatory bowel diseases
LM: low methylated
OTU: operational taxonomic unit
PCoA: principal coordinate analysis
POS: pectic oligosaccharides
PULs: polysaccharide utilization loci
QIIME: quantitative insight into microbial ecology software
RGI: rhamnogalacturonan I
Rha: rhamnose
rRNA: ribosomal RNA
SCFA: short chain fatty acids
SIEM: simulated ileal efflux medium
TIM-2: colon model
Xyl: xylose
