# Supplementary material for: Potential of Pectins to Beneficially Modulate the Gut Microbiota Depends on Their Structural Properties
Source: Front Microbiol. 2019 Feb 15;10:223. doi: 10.3389/fmicb.2019.00223 (PMC6384267; doi:10.3389/fmicb.2019.00223)
Supplement: Supplementary file 1 [file Table_1.docx]

**Supplementary Table S1.**  Composition of pectins used in this study ^1^

| **Pectin ID** | **GalA, %** | **Gal, %** | **Ara, %** | **Rha, %** | **Glc, %** | **Xyl, %** | **HG, %** | **RG, %** | **DBr, %** |
| --- | --- | --- | --- | --- | --- | --- | --- | --- | --- |
| P1 | 66.2 ^bc^ (1.9) | 18.7 ^abc^ (0.7) | 2.81 ^d^ (0.41) | 2.64 ^bcd^ (0.19) | 8.73 ^a^ (0.48) | 0.63 ^bc^ (0.03) | 63.5 ^bc^ (1.6) | 26.8 ^c^ (2.0) | 14.9 ^ab^ (0.3) |
| P2 | 73.1 ^ab^ (2.2) | 14.8 ^bc^ (0.6) | 7.10 ^bcd^ (0.21) | 1.79 ^cd^ (0.12) | 1.98 ^bc^ (0.11) | 0.25 ^d^ (0.01) | 71.3 ^b^ (0.5) | 25.5 ^c^ (1.9) | 10.8 ^b^ (0.2) |
| P3 | 70.7 ^b^ (1.7) | 10.4 ^c^ (0.4) | 15.2 ^bc^ (0.2) | 1.50 ^d^ (0.19) | 1.14 ^c^ (0.20) | 0.29 ^d^ (0.01) | 69.2 ^b^ (0.2) | 28.6 ^c^ (1.5) | 7.4 ^b^ (1.0) |
| P4 | 46.9 ^d^ (4.0) | 27.9 ^ab^ (1.8) | 16.7 ^b^ (1.4) | 3.05 ^abc^ (0.12) | 3.66 ^bc^ (0.19) | 0.51 ^bc^ (0.02) | 43.9 ^d^ (2.3) | 50.7 ^b^ (2.7) | 7.9  ^b^ (1.0) |
| P5 | 59.9 ^c^ (0.6) | 26.8 ^ab^ (0.7) | 2.80 ^d^ (0.09) | 3.72 ^ab^ (0.47) | 4.55 ^b^ (0.16) | 0.76 ^b^ (0.08) | 56.2 ^c^ (1.0) | 37.1 ^bc^ (2.6) | 15.6 ^ab^ (1.6) |
| P6 | 82.0 ^a^ (7.1) | 9.07 ^c^ (0.93) | 4.25 ^cd^ (0.29) | 1.77 ^cd^ (0.13) | 0.70 ^c^ (0.29) | 0.21 ^d^ (0.01) | 81.3 ^a^ (3.6) | 16.8 ^d^ (1.2) | 19.2 ^a^ (2.8) |
| P7 | 67.3 ^bc^ (1.9) | 22.0 ^abc^ (0.5) | 3.14 ^cd^ (0.17) | 2.86 ^bcd^ (0.24) | 2.71 ^bc^ (0.18) | 0.39 ^cd^ (0.02) | 64.5 ^bc^ (1.1) | 30.8 ^c^ (0.3) | 15.1 ^ab^ (2.5) |
| P8 | 52.9 ^cd^ (0.3) | 30.7 ^a^ (2.6) | 2.80 ^d^ (0.14) | 2.41 ^bcd^ (0.34) | 8.48 ^a^ (0.70) | 0.80 ^b^ (0.07) | 50.5 ^cd^ (0.2) | 38.3 ^bc^ (2.6) | 8.3 ^b^ (0.1) |
| P9 | 72.9 ^ab^ (2.2) | 21.2 ^abc^ (0.4) | 0.82 ^d^ (0.22) | 1.72 ^cd^ (0.15) | 1.52 ^c^ (0.31) | 0.49 ^bcd^ (0.02) | 71.2 ^b^ (0.6) | 25.5 ^c^ (1.8) | 10.4 ^b^ (1.8) |
| P10 | 11.0 ^e^ (1.1) | 7.92 ^c^ (4.44) | 69.9 ^a^ (5.0) | 6.62 ^a^ (0.98) | 1.67 ^bc^ (0.58) | 1.78 ^a^ (0.09) | 4.41 ^e^ (2.72) | 91.1 ^a^ (2.5) | 8.5 ^b^ (1.2) |

^1^ Galacturonic acid (GalA), arabinose (Ara), rhamnose (Rha), galactose (Gal), glucose (Glc) and xylose (Xyl) were quantified HPAEC and presented by mean values and SD (in brackets) from two repeats. Calculated values: homogalacturonan, HG% = GalA% – Rha%, rhamnogalacturonan, RG% = 2Rha% + Ara% + Gal%, and degree of branching DBr% = 100 % × Rha%/(Ara% + Gal%). The properties of pectins have been reported previously (Larsen et al., 2018). Statistical differences (except for DBr) were analyzed in this study. Superscripts (a, b, c, d, and e) within a column show significant differences (p < 0.05) between the pectins, determined by one-way ANOVA, Tukey's post-hoc test.
