# Supplementary material for: Potential of Pectins to Beneficially Modulate the Gut Microbiota Depends on Their Structural Properties
Source: Front Microbiol. 2019 Feb 15;10:223. doi: 10.3389/fmicb.2019.00223 (PMC6384267; doi:10.3389/fmicb.2019.00223)
Supplement: Supplementary file 2 [file Table_2.docx]

**Supplementary Table S2.** Cumulative production of branched chain fatty acids (BCFA) iso-butyrate and iso-valerate after 72 h fermentation of pectins in TIM-2 colon model ^1^

| **Pectin ID** | **iso-butyrate, mmol** | **iso-valerate, mmol** | **Total BCFA, mmol** |
| --- | --- | --- | --- |
| P1 | 5.1 (0.8) | 17.9 (3.1) | 23.1 (3.9) |
| P2 | 4.0 (0.6) | 15.4 (0.8) | 19.4 (0.2) |
| P3 | 5.0 (0.9) | 14.7 (0.5) | 19.6 (1.4) |
| P4 | 5.1 (0.7) | 13.1 (0.6) | 18.2 (1.3) |
| P5 | 5.0 (0.1) | 14.4 (0.6) | 19.4 (0.7) |
| P6 | 4.7 (0.8) | 14.2 (0.4) | 18.9 (1.2) |
| P7 | 4.8 (0.3) | 15.9 (0.8) | 20.7 (1.2) |
| P8 | 4.8 (0.5) | 15.4 (2.3) | 20.2 (2.8) |
| P9 | 4.0 (0.3) | 13.8 (1.9) | 17.8 (2.2) |
| P10 | 2.9 (0.9) | 9.4 (1.4) | 12.3 (1.9) |

^1^ Mean values of BCFA and SD (in brackets) in lumen and dialysate samples (combined data) from two independent TIM-2 experiments. Total BCFA is a sum of iso-butyrate and iso-valerate. Pectin IDs are explained in Table 1. The amount of BCFA at the start of fermentations has been set to zero.
