# Supplementary material for: Potential of Pectins to Beneficially Modulate the Gut Microbiota Depends on Their Structural Properties
Source: Front Microbiol. 2019 Feb 15;10:223. doi: 10.3389/fmicb.2019.00223 (PMC6384267; doi:10.3389/fmicb.2019.00223)
Supplement: Supplementary file 3 [file Table_3.docx]

**Supplementary Table S3.** Mapping file for the metadata deposited in the European Nucleotide Archive (ENA) database.

| **SampleID^1^** | **Pectin ID** | **Time, h** | **Repeat^2^** | **Counts^3^** |  | **SampleID^1^** | **Pectin ID** | **Time, h** | **Repeat^2^** | **Counts^3^** |
| --- | --- | --- | --- | --- | --- | --- | --- | --- | --- | --- |
| NL.049 | P1 | 0 | 1 | 62587 |  | NL.065 | P5 | 0 | 1 | 73747 |
| NL.001 | P1 | 0 | 2 | 42594 |  | NL.017 | P5 | 0 | 2 | 61126 |
| NL.050 | P1 | 24 | 1 | 56691 |  | NL.066 | P5 | 24 | 1 | 120500 |
| NL.002 | P1 | 24 | 2 | 41690 |  | NL.018 | P5 | 24 | 2 | 37089 |
| NL.051 | P1 | 48 | 1 | 91082 |  | NL.067 | P5 | 48 | 1 | 114801 |
| NL.003 | P1 | 48 | 2 | 36941 |  | NL.019 | P5 | 48 | 2 | 87903 |
| NL.193 | P1 | 56 | 1 | 22427 |  | NL.197 | P5 | 56 | 1 | 224047 |
| NL.117 | P1 | 56 | 2 | 96003 |  | NL.121 | P5 | 56 | 2 | 59564 |
| NL.052 | P1 | 72 | 1 | 92298 |  | NL.068 | P5 | 72 | 1 | 101029 |
| NL.004 | P1 | 72 | 2 | 30074 |  | NL.020 | P5 | 72 | 2 | 70655 |
| NL.085 | P10 | 0 | 1 | 41551 |  | NL.069 | P6 | 0 | 1 | 71661 |
| NL.037 | P10 | 0 | 2 | 43848 |  | NL.021 | P6 | 0 | 2 | 84570 |
| NL.086 | P10 | 24 | 1 | 55792 |  | NL.070 | P6 | 24 | 1 | 76940 |
| NL.038 | P10 | 24 | 2 | 62027 |  | NL.022 | P6 | 24 | 2 | 79041 |
| NL.087 | P10 | 48 | 1 | 66102 |  | NL.071 | P6 | 48 | 1 | 68871 |
| NL.039 | P10 | 48 | 2 | 56997 |  | NL.023 | P6 | 48 | 2 | 66331 |
| NL.202 | P10 | 56 | 1 | 51037 |  | NL.198 | P6 | 56 | 1 | 167479 |
| NL.126 | P10 | 56 | 2 | 122861 |  | NL.122 | P6 | 56 | 2 | 85351 |
| NL.088 | P10 | 72 | 1 | 44661 |  | NL.072 | P6 | 72 | 1 | 55262 |
| NL.040 | P10 | 72 | 2 | 98307 |  | NL.024 | P6 | 72 | 2 | 49971 |
| NL.053 | P2 | 0 | 1 | 135278 |  | NL.073 | P7 | 0 | 1 | 67918 |
| NL.005 | P2 | 0 | 2 | 56450 |  | NL.025 | P7 | 0 | 2 | 60073 |
| NL.054 | P2 | 24 | 1 | 193948 |  | NL.074 | P7 | 24 | 1 | 84180 |
| NL.006 | P2 | 24 | 2 | 44340 |  | NL.026 | P7 | 24 | 2 | 78305 |
| NL.055 | P2 | 48 | 1 | 170077 |  | NL.075 | P7 | 48 | 1 | 73186 |
| NL.007 | P2 | 48 | 2 | 54358 |  | NL.027 | P7 | 48 | 2 | 63755 |
| NL.194 | P2 | 56 | 1 | 139856 |  | NL.199 | P7 | 56 | 1 | 110577 |
| NL.118 | P2 | 56 | 2 | 74214 |  | NL.123 | P7 | 56 | 2 | 101950 |
| NL.056 | P2 | 72 | 1 | 107706 |  | NL.076 | P7 | 72 | 1 | 107336 |
| NL.008 | P2 | 72 | 2 | 43063 |  | NL.028 | P7 | 72 | 2 | 84888 |
| NL.057 | P3 | 0 | 1 | 57757 |  | NL.077 | P8 | 0 | 1 | 74831 |
| NL.009 | P3 | 0 | 2 | 51545 |  | NL.029 | P8 | 0 | 2 | 112305 |
| NL.058 | P3 | 24 | 1 | 40228 |  | NL.078 | P8 | 24 | 1 | 87239 |
| NL.010 | P3 | 24 | 2 | 48083 |  | NL.030 | P8 | 24 | 2 | 22069 |
| NL.059 | P3 | 48 | 1 | 76032 |  | NL.079 | P8 | 48 | 1 | 85110 |
| NL.011 | P3 | 48 | 2 | 12512 |  | NL.031 | P8 | 48 | 2 | 100434 |
| NL.119 | P3 | 56 | 2 | 59258 |  | NL.200 | P8 | 56 | 1 | 89707 |
| NL.218 | P3 | 56 | 1 | 12802 |  | NL.124 | P8 | 56 | 2 | 123167 |
| NL.060 | P3 | 72 | 1 | 51537 |  | NL.080 | P8 | 72 | 1 | 72148 |
| NL.012 | P3 | 72 | 2 | 43305 |  | NL.032 | P8 | 72 | 2 | 90819 |
| NL.061 | P4 | 0 | 1 | 59605 |  | NL.081 | P9 | 0 | 1 | 77136 |
| NL.013 | P4 | 0 | 2 | 58378 |  | NL.033 | P9 | 0 | 2 | 89510 |
| NL.062 | P4 | 24 | 1 | 72908 |  | NL.082 | P9 | 24 | 1 | 79085 |
| NL.014 | P4 | 24 | 2 | 70682 |  | NL.034 | P9 | 24 | 2 | 86076 |
| NL.063 | P4 | 48 | 1 | 74477 |  | NL.083 | P9 | 48 | 1 | 79555 |
| NL.015 | P4 | 48 | 2 | 80879 |  | NL.035 | P9 | 48 | 2 | 75846 |
| NL.196 | P4 | 56 | 1 | 64478 |  | NL.201 | P9 | 56 | 1 | 179659 |
| NL.120 | P4 | 56 | 2 | 40233 |  | NL.125 | P9 | 56 | 2 | 114386 |
| NL.064 | P4 | 72 | 1 | 65340 |  | NL.084 | P9 | 72 | 1 | 51517 |
| NL.016 | P4 | 72 | 2 | 78184 |  | NL.036 | P9 | 72 | 2 | 44505 |

^1^Sample ID as uploaded to ENA database [accession number: PRJEB25646];^2^ Independent TIM-2 fermentation ^3^ No. of reads obtained per sample using NextSeq 550 Illumina DNA amplicon sequencing platform (V3 region of 16S rRNA gene).
