# Supplementary figures and images for: Potential of Pectins to Beneficially Modulate the Gut Microbiota Depends on Their Structural Properties
Source: Front Microbiol. 2019 Feb 15;10:223. doi: 10.3389/fmicb.2019.00223 (PMC6384267; doi:10.3389/fmicb.2019.00223)

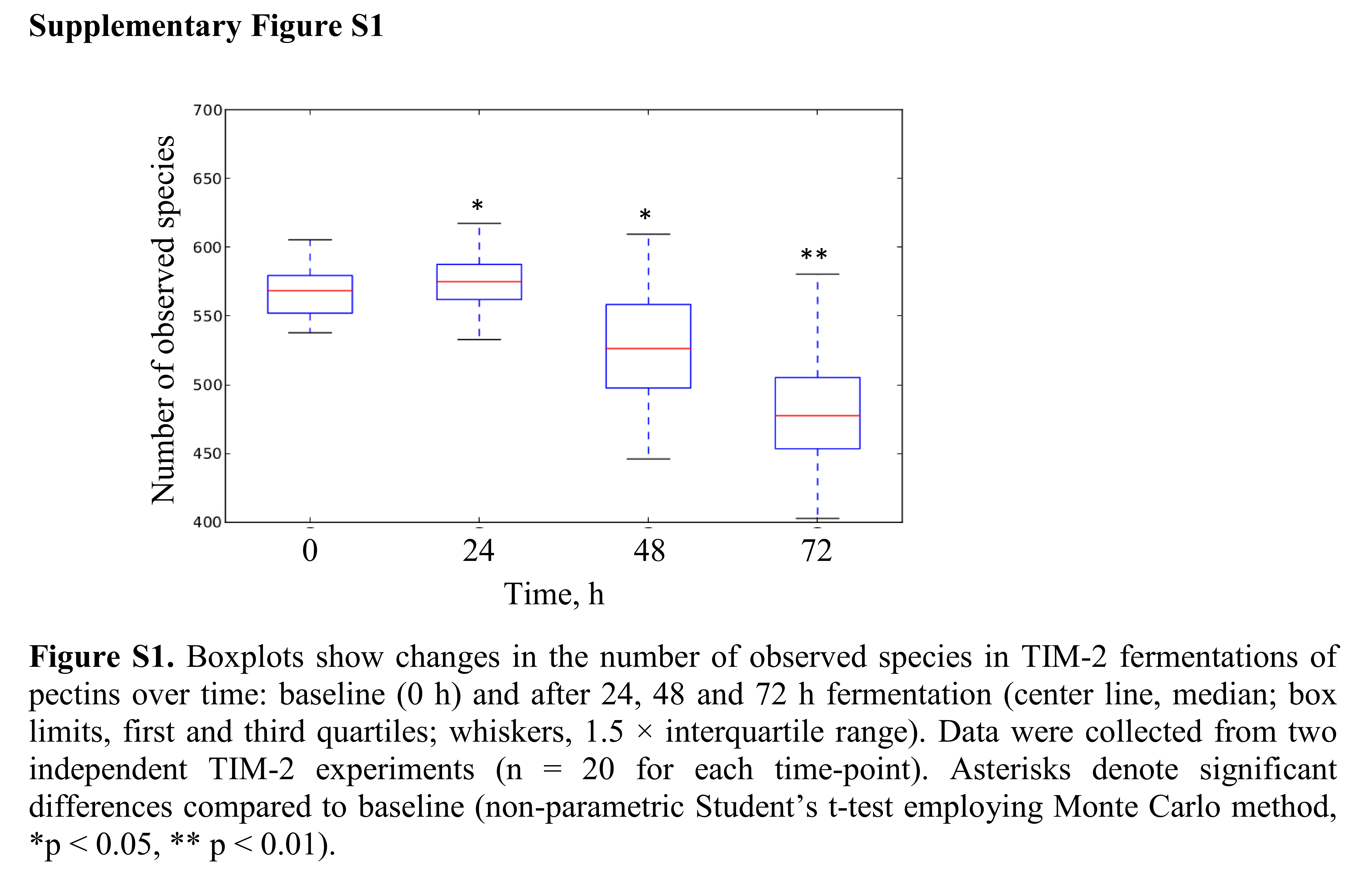

Supplement: Supplementary file 4 [file Image_1.tiff]
