# Supplementary material for: Potential of Pectins to Beneficially Modulate the Gut Microbiota Depends on Their Structural Properties
Source: Front Microbiol. 2019 Feb 15;10:223. doi: 10.3389/fmicb.2019.00223 (PMC6384267; doi:10.3389/fmicb.2019.00223)
Supplement: Supplementary file 5 [file Image_2.pdf]

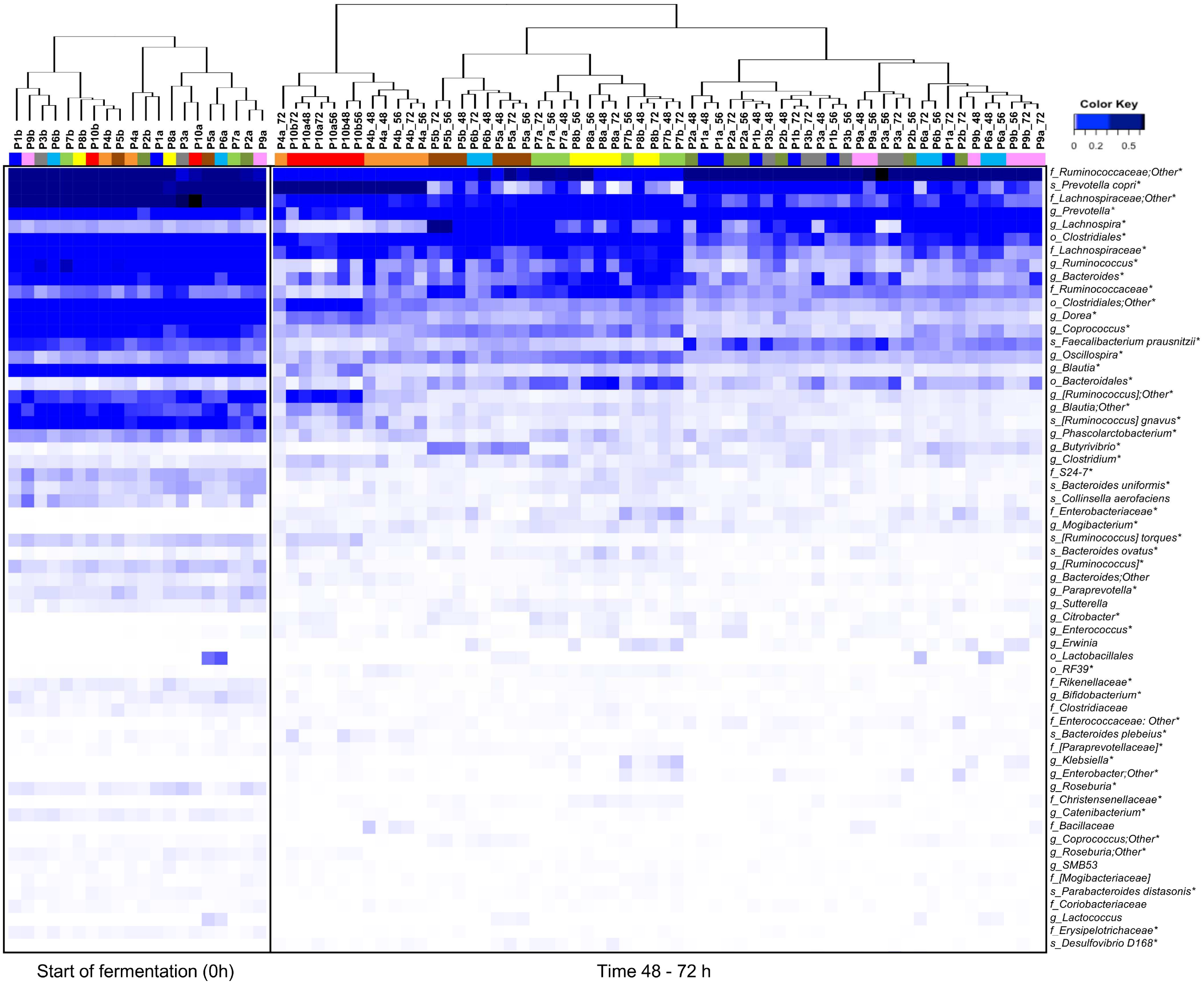

Figure S2. Heatmap showing relative abundances of species level OTUs (cut-off 0.01%) at start of fermentation (0 h) and after 48, 56 and 72 h fermentation of pectins in TIM-2 colon model. Hierarchical clustering of pectins was performed by R-studio software heatmap2. Sample codes include pectin ID (P1 – P10), experimental repeat (a or b) and fermentation time (48, 56 and 72). Bacterial taxa significantly changed in fermentations are marked by asterisks. Differences were analyzed for combined data set (48, 56 and 72 h) compared to time 0 h (pooled data, n = 20) obtained in two independent experiments (n = 6 for each pectin), using the Wilcoxon Rank Sum test and Bonferroni multiplicity correction ($p < 0.05$). Taxa denoted as “Other” indicated that more than one taxon could be assigned to this cluster at given taxonomic level. Taxa in square brackets indicated a proposed taxonomy.
